# Age-Related Impairment of Pancreatic Beta-Cell Function: Pathophysiological and Cellular Mechanisms

**DOI:** 10.3389/fendo.2014.00138

**Published:** 2014-09-03

**Authors:** Vincenzo De Tata

**Affiliations:** ^1^Department of Translational Research and New Technologies in Medicine and Surgery, University of Pisa, Pisa, Italy

**Keywords:** aging, pancreatic beta cells, insulin secretion, insulin resistance, cellular senescence

## Abstract

The incidence of type 2 diabetes significantly increases with age. The relevance of this association is dramatically magnified by the concomitant global aging of the population, but the underlying mechanisms remain to be fully elucidated. Here, some recent advances in this field are reviewed at the level of both the pathophysiology of glucose homeostasis and the cellular senescence of pancreatic islets. Overall, recent results highlight the crucial role of beta-cell dysfunction in the age-related impairment of pancreatic endocrine function and delineate the possibility of new original therapeutic interventions.

## Type 2 Diabetes: An Age-Related Disease

Diabetes mellitus, a disease characterized by high blood glucose levels resulting from a combination of genetic and acquired factors, represents the most prevalent metabolic disorders. Indeed, the prevalence of the most common form, type 2 diabetes or non-insulin-dependent diabetes mellitus (NIDDM), exploded over the last several decades. Data from the World Health Organization (WHO) and the International Diabetes Federation (IDF) show that the prevalence of type 2 diabetes increased from 100–135 million affected adults worldwide in 1994–1995 to approximately 336 million in 2011, and it is expected to rise to 439 million by 2030 (1–3). These data are even more dramatic considered in the light of the concomitant global aging of the population. Elderly people (by definition, person of over 65 years of age) represented 12–15% of the population in 2008, whereas it has been estimated that the same age group would account for 26% of the population in 2026 and will reach 2 billion people in 2050 (4, 5). Population aging is rapidly becoming a global issue with a major impact on health policies and programs. Such a remarkable improvement in life expectancy considerably contributed to a shift in the leading causes of diseases and death from infectious and parasitic diseases to non-communicable diseases (such as heart disease, cancer, and diabetes) that more commonly affect adults and older adults (6, 7). In particular, aging is an important risk factor for metabolic disorders, including obesity, impaired glucose tolerance, and type-2 diabetes (8, 9). The prevalence of type 2 diabetes increases with age (in older adults it is more than twice that of middle-aged adults) and peaks at 60–74 years of age (10–12). In consideration of the already mentioned nearly doubling of the numbers of elderly persons by the year 2030, it is easy to see why diabetes in older adults is considered as a growing public health concern.

Normal aging is usually associated with a progressive deterioration in most endocrine functions that may be responsible for serious disturbances of metabolic homeostasis (13–16). Actually, an impairment of glucose tolerance has been recognized for a long time as a well-known feature of aging in both humans and experimental animals (17, 18). Nevertheless, the underlying biological mechanism(s) is still not clearly understood.

## Aging and Insulin Resistance

Type 2 diabetes mellitus is a metabolic disorder characterized by high blood glucose levels as a result of the complex interplay of multiple genetic and environmental factors that cause both impaired insulin action on target tissues and defective pancreatic beta-cell insulin secretion in response to glucose (19).

Traditional views of type 2 diabetes pathophysiology indicated peripheral insulin resistance, i.e., the inability of insulin-target tissues to respond properly to the hormone, as the main driver of altered glucose homeostasis (20). Clinically, the term insulin resistance is utilized to indicate that higher-than-normal circulating levels of insulin are required to maintain normoglycemia. At the cellular level, insulin action, initiated by the binding to its cell surface receptor, involves a series of signaling cascades that can be schematically summarized as follows: receptor autophosphorylation and activation of receptor tyrosine kinase; tyrosine phosphorylation of insulin receptor substrates (IRSs) 1 and 2; activation of phosphatidylinositol 3-kinase (PI3K); activation of Akt and its downstream mediator, AS160, which stimulates the translocation of insulin-mediated GLUT4 from intracellular vesicles to the plasma membrane (21, 22).

It is well documented that aging is associated with a decline of insulin action. Studies utilizing the euglycemic hyperinsulinemic clamp technique to assess insulin effectiveness in regulating glucose transport usually stress the relevance of the diminished insulin sensitivity on target tissues in the development of age-related glucose intolerance (17, 20, 23, 24). Insulin resistance could increase with age in relation to several well-known age-related changes, such as: (i) increased adiposity (with particular reference to abdominal fat mass) (5); (ii) decreased lean muscle mass (sarcopenia) (25–27); (iii) mitochondrial dysfunctions (28–32); (iv) hormonal changes (33, 34); (v) increased oxidative stress and inflammation (35–39); (vi) changes in dietary habits (40–42); (vii) reduced physical activity (43, 44). However, it has also been claimed that these factors alone cannot fully account for the age-related glucose-intolerance (11), and other studies seem to indicate that age *per se* could be not responsible for the increased insulin resistance (45–47).

## Aging and Insulin Secretion

On the other hand, several observations clearly show that insulin resistance alone is not sufficient to lead to type 2 diabetes in the absence of a beta-cell defect associated with abnormal insulin secretion. Consequently, beta-cell dysfunction is increasingly recognized to play a fundamental role in type 2 diabetes pathophysiology (48, 49) and could represent another significant contributing factor to abnormal glucose metabolism with age (9, 50). Indeed, it has been repeatedly reported that the ability of pancreatic beta cells to maintain an insulin secretory function adequate for metabolic demand is impaired with increasing age in both experimental animals (51–55) and humans (11, 56–65), although some of these studies (especially in humans) were characterized by a significant degree of variability (66).

This age-related impairment of beta-cell secretory capabilities has been variously attributed to several factors, including: (i) mitochondrial dysfunction (34, 67–69); (ii) reduced GLUT2 levels (54, 70); (iii) accumulation of advanced glycation end products (AGEs) (71, 72); (iv) telomerase deficiency and reduced telomere length (73, 74): (v) reduced expression of β_2_-adrenergic receptors (75); (vi) impaired Ca^++^ handling (76, 77); (vii) reduced response to GLP-1 stimulation (62, 65, 78–83); (vii) increased autophagy (84); (viii) reduced expression of beta-cell-specific genes and transcription factors such as PDX-1 (54).

Among the above mentioned factors, mitochondrial dysfunction may deserve a particular discussion because mitochondria play a crucial role in the physiological stimulus-secretion coupling in beta cells. In these cells, mitochondria serve as nutrient sensors and signal generators for insulin secretion. In particular, the mitochondrial metabolism of pyruvate, glycolitically derived from glucose, generates ATP, which in turn promotes the closure of ATP-sensitive K^+^ channels and the consequent cell depolarization, inducing Ca^2+^ influx through voltage-gated Ca^2+^ channels, increased cytosolic [Ca^2+^], and finally triggering insulin exocytosis (85). On the other hand, due to the central role played in the generation of reactive oxygen species (ROS) at the level of the electron transport chain and ATP production, it has been proposed that mitochondria could represent a primary target of ROS damage (mitochondrial free radical theory of aging) (86). Indeed, increasing evidence suggests that abnormal mitochondrial ROS production and detoxification contribute to mitochondrial dysfunction in old age (87). Thus, age-related impairment of mitochondrial function could easily result in decreased beta-cell function and insulin secretion (88).

We can tentatively conclude this brief survey of the pathophysiology of glucose homeostasis by observing that several risk factors for diabetes associated with aging likely contribute to the development of age-related glucose intolerance and insulin resistance. Adaptation to insulin resistance normally requires compensatory hyperinsulinemia to maintain normal glucose metabolism. On the average, many studies show that, when considered in light of the degree of insulin resistance, all the indexes of insulin secretion appear to be decreased with age, indicating decreased beta-cell secretory reserve. Thus, the main homeostatic defect could be ascribed to age-dependent failure of the endocrine pancreas to provide enough insulin to overcome the state of increased peripheral insulin resistance.

## Beta-Cell Senescence

Studies on the age-related glucose intolerance at the pathophysiological level may be difficult to interpret because the development of this condition could depend on a combination of many different factors whose independent influence is not easily controlled, thus making their relative importance a matter of debate. Therefore, more recently several researchers shifted the focus of their interest on the effect of aging on islet biology, with particular reference to the proliferative and regenerative capacity of beta cells. This paradigmatic change arises mainly from the consideration that aging represents a major risk factor for many generally chronic diseases (including cancer, neurodegeneration, and diabetes) and from the related possibility that these pathologies could be linked by a common biology. In the last few decades, a growing consensus has been reached and now it is considered likely that one or more basic aging processes underlie most, if not all, age-related pathologies (89). One basic process that may contribute to age-related dysfunction, including decreased secretory function (90), is cellular senescence. Cellular senescence was firstly described more than 50 years ago by Hayflick and Moorhead (91) as a process limiting the proliferation of normal human fibroblasts in culture, and this term is now generally used to indicate the essentially irreversible growth arrest that occurs when cells that can divide are challenged by a potentially oncogenic stress (92, 93). Senescent cells have clearly been shown to disrupt normal tissue structures and differentiated functions in complex cell culture models (89).

The growing interest in the cellular mechanisms responsible for the age-related decline in beta-cell proliferation originated from two distinct considerations with either fundamental or clinical implications. (A) Since insulin secretion by pancreatic beta cells represents the key point of the endocrine axis regulating glucose homeostasis, it is obvious that maintenance of beta-cell number and islet mass must be considered crucial in order to sustain normoglycemia. (B) Beta-cell replication represents a major goal of the cellular therapy of diabetes. Indeed, the promising attempt to develop a therapy based on pancreatic islets transplantation is still seriously hampered by the scarcity of cadaver-derived islets. The possibility to enhance replication of islet cells *in vitro* has been proposed as a solution to overcome the limited supply. Similarly, the expansion of potentially reduced functional beta-cell mass *in vivo* might represent another therapeutic strategy in type 1 and type 2 diabetes.

In normal healthy conditions, beta cells have a long lifespan with a low proliferation rate (94). However, it has been shown that in particular conditions, such as in response to increased metabolic demand or after injury, the adult pancreas could be able to produce new cells, particularly beta cells. Recent experimental evidences indicate that beta-cell mass, like many other tissues, could be dynamically regulated with ongoing beta-cell regeneration throughout life to replace lost or damaged beta cells (95).

## Molecular Mechanism of Age-Related Beta-Cell Growth Arrest

**Figure 1:**
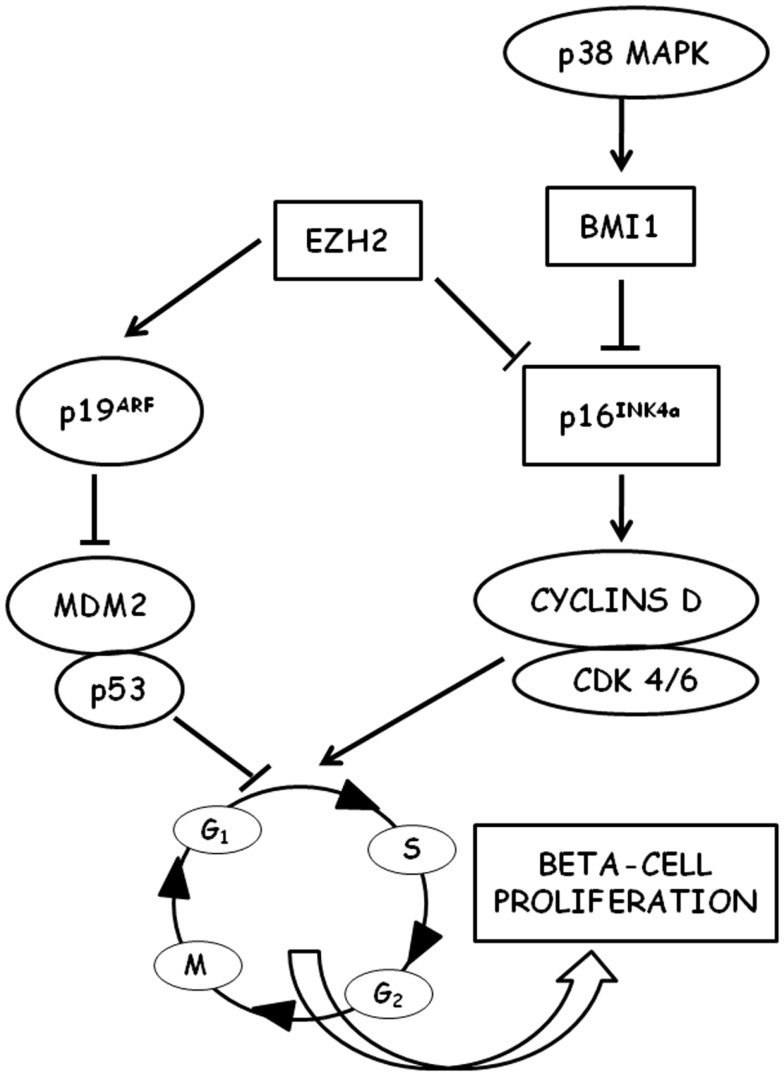
**Schematic representation of the molecular pathways involved in the regulation of beta-cell proliferation is shown**. P16^INK4a^ is a key regulator of cell cycle entry in aged beta cells through D-type cyclins and cyclin-dependent kinases (CDK). P16^INK4a^ is negatively regulated by the polycomb proteins EHZ3 (enhancer of zeste homolog 2) and BMI1 (B lymphoma Mo-MLV insertion region 1 homolog). BMI1 is stimulated by p38 MAPK.

Beta-cell cycling is driven by cyclin D1/D2-Cdk activity and is repressed by the Cdk-inhibitor p^16INK4a^ (Figure 1) (96). In mice, it has been shown that beta-cell proliferation is an age-related process and that the expansion of beta-cell mass after pancreatic injury is more robust in young than in old animals (97). However, several pieces of experimental evidence indicate that aging mouse beta cells maintain a partially preserved ability to proliferate when specifically stimulated, both after pancreas injury (such as partial pancreatectomy or beta-cell-specific cell ablation) (98–102) and after islet transplantation in hyperglycemic recipients (103, 104). On the other hand, in recent years it became increasingly apparent that many of the mechanisms identified in these rodent models cannot be transferred easily to human islet cells. Human studies generally consist of observations made from pancreases obtained at autopsy, pancreas donation, and surgical resection, and are mainly based on immunohistochemical markers of proliferation (such as the nuclear Ki-67). As a consequence, data obtained in humans are often less conclusive than those obtained in rodent experimental models (105). It has been shown that human beta-cell mass can increase in obesity, although to a lesser degree than in rodents (30–40% estimated increase in humans with respect to a 30-fold increase observed in mice) (106–108). On the contrary, recent studies failed to detect an increased rate of beta-cell proliferation in pregnant individuals and in type 2 diabetes patients (109).

A major difference between mice and humans is telomere shortening that limits proliferation and leads to cellular senescence in humans (110, 111), whereas in mice that have long telomeres no impairment of replication has been detected for several generations after ablation of telomerase (112, 113). This difference may account for the differential response observed between mice and humans (proliferation vs. differentiation from non-beta-cell progenitors) in beta-cell compensation (114). Human beta cells in adults appear to be largely postmitotic with very low rates of cell proliferation after the age of 20–30 years, as determined by Ki-67 content (115–117), thymidine analog incorporation (118), and increased *in vivo* lipofuscin accumulation (119, 120). Growth arrest of adult human beta cells cannot be reversed by procedures inducing proliferation *in vitro* (121, 122). This decline in the proliferative capacity of aging beta cells is directly associated with a decreased expression of the pancreatic and duodenal homeobox 1 (Pdx1) (121, 123), a transcription factor that plays a crucial role in beta-cell replication (124). Several experimental pieces of evidence demonstrated a decreased expression of cell cycle activators (such as, e.g., the transcription factor FoxM1) in aging beta cells with a simultaneous decrease in the expression of cell cycle inhibitors [for a review see Ref. (10)]. p16^INK4a^ tumor suppressor protein has emerged from these studies as a key control point for cell cycle entry of beta cells. p16^INK4a^ is a cyclin-dependent kinase inhibitor (CDKI) encoded by the Cdkn2a locus, which sequesters cdk4 and cdk6, thus preventing their interaction with the D cyclins. It has been shown that p16^INK4a^ expression increases with age in several mouse tissues, including islets (125), and that proliferation of beta cells in young mice was reduced to levels observed in older mice when the transgenic overexpression of p16^INK4a^ was induced (125, 126). On the other hand, in p16^INK4a^ knockout mice, beta-cell proliferation was significantly increased (126). In this context, it could be very intriguing to mention that genome-wide association studies revealed an association between SNPs near Cdk2a (the locus encoding p16^INK4a^) and increased risk of type 2 diabetes (113, 127, 128). It has also been shown that free fatty acids, whose levels were typically increased in type 2 diabetes and that could be responsible for beta-cell damage (129), can induce p16^INK4a^ expression in islets (130). Thus, p16^INK4a^ could represent a potential link between aging, metabolic derangements, and beta-cell failure in type 2 diabets (131). More recently, it has been shown that the age-associated decrease in p16^INK4a^ expression in pancreatic islets could be related to the decreased expression of BMI1 (132) and EZH2 (133), two chromatin-regulating polycomb group proteins, indicating the crucial role that epigenetic regulation could play in the control of cell cycle progression of beta cells in both aging and type 2 diabetes (134). Indeed, mice with conditional gene inactivation of EZH2 in beta cells exhibited a premature increase in p16^INK4a^ and p19^arf^ expression and a reduced beta-cell proliferation, whereas no changes were observed in the levels of other CDK inhibitors, suggesting a specific effect of EZH2 on the INK4a/arf locus in beta cells (133). However, the transgenic expression of EZH2 was unable to repress INK4a in mice older than 8 months, unless EZH2 was expressed in conjunction with knockdown of trithorax group (TrxG) protein complex components (135).

Overall, these results indicate that cellular senescence could be responsible for the observed decline in the proliferative capacity of pancreatic beta cells. It has been reported that Akita mice with short telomeres are characterized by slower proliferation of beta cells and accumulation of p16^INK4a^ (74). More recently, Zeng et al. (136) showed that in mice the beta-cell-specific genetic deletion of Pten (phosphatase and tensin homolog), encoding a tumor suppressor protein involved in the regulation of the cell cycle (137), prevents the age-related decline in beta-cell proliferation and restores the ability of beta cells to respond to injury-mediated regeneration. Interestingly, the ability of Pten deletion to remove the block in cell cycle re-entry seems to be mediated by a decrease in p16^INK4a^ expression.

The decline in beta-cell proliferation with age may also be the result of an age-related impairment of mitotic signal transduction pathways. It has been shown that p38 MAPK signals are able to influence CDKI expression in aged islets: the destruction of p38 MAPK signals in aged mutant mice has as a consequence a reduced expression of p16^INK4a^, p19^arf^, and other CDKI with a related increase of beta-cell proliferation (138). This effect seems to be counterbalanced by the p53-induced phosphatase 1 (WIP1), whose overexpression in middle-aged transgenic mice causes a reduced p16^INK4a^ expression as well as an improved capacity of beta-cell regeneration after selective beta-cell destruction by streptozotocin (138). A further important component linking growth signals to beta-cell expansion could likely be represented by Akt activation and its downstream mTORC1 signaling (137). It is well known that alterations in the nutrient-sensing pathways (such as the insulin/IGF-1 and the TOR pathways) have been proposed to underlie the aging process and modulate longevity (139). mTOR is an evolutionarily conserved nutrient-sensing cytoplasmic protein kinase that regulates cell growth and metabolism in response to mitogens, nutrients, and hormones in all eukaryotic cells (140). However, later in life, when growth has been completed, mTOR can drive cellular and organismal aging (141) and can be involved in age-related diseases (138). Indeed, the most well-known TOR inhibitor, rapamycin, is able to extend lifespan in yeast, flies, worms, and rodents (142). Glucose, amino acids, and fatty acids activate mTOR in beta cells, and the consequent increase in beta-cell mass and function may help to compensate the age-related development of insulin resistance (143). However, it has been proposed that, during aging, the chronic hyperstimulation of mTOR could contribute to the development of beta-cell failure (143). Interestingly, metformin, the most widely used anti-diabetic drug, has been shown to be an inhibitor of mTORC1 and to decrease the phosphorylation of its substrates S6K1 and 4E-BP1 (144). Metformin was also shown to increase longevity in species ranging from yeast to mice (145). The underlying mechanism of this action of metformin is not fully understood. However, it is known that metformin inhibits the activity of mitochondrial complex I and increases the activity of AMPK, which in turn inhibits mTORC1 complex activity, thus suggesting a possible link between rapamycin and metformin actions on longevity.

Little is known about the upstream signals that could be responsible for the regulation of beta-cell proliferation and its decline with age. It has been reported that PDGF treatment increased beta-cell proliferation in cultured human islets from young donors but not in islets from adults. Interestingly, PDGF receptor signals seem to act in part via EZH2 (146). Treatment with the glucagon-like peptide 1 (GLP-1) analog, exendin-4 is able to increase beta-cell mass and markedly decrease p16^INK4a^ expression in young but not in middle-aged mice (147). Recently, it has been shown with parabiosis experiments that a systemic factor (whose exact nature is still unknown) found in the circulation of young mice seems to be able to increase the proliferation rate of old pancreatic beta cells (148).

## Conclusion

**Figure 2:**
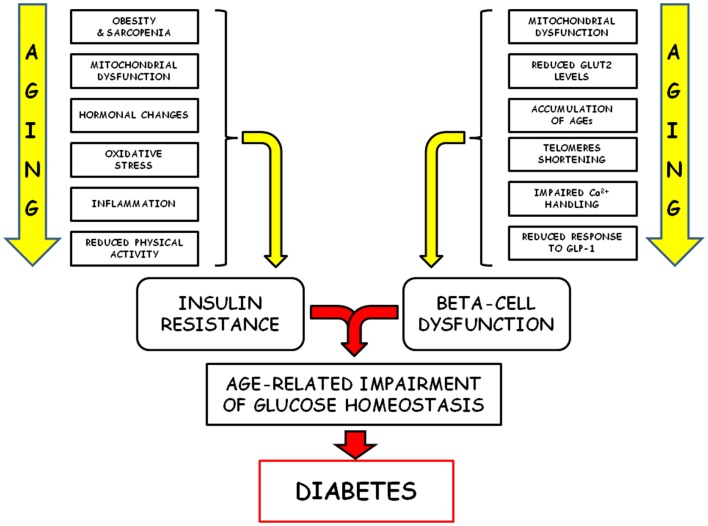
**Schematic representation of the pathophysiological factors responsible of the age-related failure of glucose homeostasis is shown**.

Alterations of glucose homeostasis increase with age and represent leading causes of morbidity and mortality, mainly linked to both the complications associated with type 2 diabetes and the increased risk for several other age-related diseases (149). The classical pathophysiological factors responsible for this age-related failure of glucose homeostasis (insulin resistance and decreased secretory capability of beta cells) are quite well characterized, but new mechanisms have recently been revealed (Figure 2). Central to this new development is the key concept that loss or dysfunction of pancreatic beta cells plays a crucial role in the pathogenesis of type 2 diabetes. Since the predominant mechanism of beta-cell generation seems to be self-renewal, the senescence-associated cell cycle dysregulation and the consequent proliferative arrest assume a particular relevance. In recent years, some of the cellular and molecular mechanisms associated with the decreased proliferation capability of senescent beta cells have been explored, but some others remain to be fully elucidated, and a further effort will be requested in order to efficiently translate this new insight into successful new therapeutic strategies.

## Conflict of Interest Statement

The author declares that the research was conducted in the absence of any commercial or financial relationships that could be construed as a potential conflict of interest.
